# In Vitro Comparison of Void Formation Between Calcium Silicate-Based Sealers and AH Plus: A Systematic Review and Meta-Analysis

**DOI:** 10.3390/dj14080496

**Published:** 2026-08-07

**Authors:** Péter Komora, Orsolya Vámos, Gergely Agócs, Boglárka Lilla Szentes, Péter Hegyi, Eszter Ágnes Szalai, Alexander Schulze Wenning, Gábor Varga, János Vág, Beáta Kerémi

**Affiliations:** 1 Department of Restorative Dentistry and Endodontics, Semmelweis University, 1088 Budapest, Hungary; komora.peter@semmelweis.hu (P.K.);; 2 Centre for Translational Medicine, Semmelweis University, 1085 Budapest, Hungary; 3 Department of Prosthodontics, Semmelweis University, 1088 Budapest, Hungary; 4 Department of Biophysics and Radiation Biology, Semmelweis University, 1094 Budapest, Hungary; 5 Institute of Pancreatic Diseases, Semmelweis University, 1083 Budapest, Hungary; 6 Institute for Translational Medicine, Medical School, University of Pécs, 7624 Pécs, Hungary; 7 Department of Oral and Maxillofacial Surgery, Regional Plastic Surgery, Dortmund General Hospital, 44145 Dortmund, Germany; 8 Department of Oral Biology, Semmelweis University, 1089 Budapest, Hungary

**Keywords:** root canal treatment, endodontic sealers, root canal sealing, biocompatible materials, evidence-based obturation

## Abstract

**Background:** Achieving a three-dimensional seal of the root canal system is considered essential for long-term endodontic success. Because no clinically acceptable threshold for void formation has been established, comparisons with clinically validated reference materials are needed to interpret the sealing quality of newer calcium silicate-based sealers (CSBSs). AH Plus is a well-established epoxy resin-based sealer (ERBS) with documented long-term clinical performance. This systematic review and meta-analysis compared the quality of root canal fillings produced with CSBSs and AH Plus using different obturation techniques in permanent mature extracted and artificial teeth. **Methods:** MEDLINE (PubMed), EMBASE, Cochrane Library, and Web of Science were systematically searched for randomized in vitro studies up to 1 July 2025. The primary outcomes were the percentages of void volume or area. Mean values and mean differences (MDs) with corresponding 95% confidence intervals (CIs) were calculated for the total filling volume and, where available, for the apical, middle, and coronal thirds of the root canal. Risk of bias and certainty of evidence were assessed. **Results:** Twenty-one studies met the eligibility criteria, and 18 articles were included in the quantitative analysis. No statistically significant differences were observed in total void volume between CSBSs placed with the single-cone technique and AH Plus used with either the single-cone or warm vertical compaction technique in extracted teeth. Similarly, no significant differences were observed between the investigated groups in the apical, middle, or coronal thirds of the root canal. Evidence for other obturation techniques and artificial teeth was limited. **Conclusions:** Within the limitations of the available in vitro evidence, no statistically significant differences in void formation were detected between the investigated sealer–technique combinations. Further standardized in vitro studies and well-designed clinical investigations are warranted.

## 1. Introduction

Achieving a hermetic three-dimensional seal of the root canal system is essential for the long-term success of endodontic treatment [1]. Voids and gaps within the root canal filling (RCF) may allow microbial leakage and compromise periapical healing [2,3,4,5]. While epoxy-resin-based sealers (ERBSs) such as AH Plus remain the gold standard, calcium silicate-based sealers (CSBSs) have gained popularity due to their bioactivity and simplified obturation protocols. However, whether these materials provide comparable filling quality remains controversial. Therefore, this systematic review and meta-analysis compared CSBSs and AH Plus with respect to void formation in in vitro studies.

This encompasses apical, lateral, and coronal sealing [6]. The apical seal prevents microbial and tissue fluid ingress from the periapical area [7,8,9]. The lateral seal adapts the filling material to canal walls, sealing dentinal tubules and anatomical irregularities while entombing residual microorganisms [10,11]. The coronal seal ensures both canal sealing and the quality of the final restoration, influencing long-term treatment outcomes [12,13].

Root canal obturation relies on gutta-percha (core material) combined with a sealer to achieve adaptation to canal walls [14]. The sealer fills interfacial gaps and accessory anatomy—particularly in the apical delta—where the core material alone cannot penetrate [15,16].

ERBSs are considered the gold standard for evaluating new endodontic sealers due to their reliable sealing performance [17]. AH Plus (Dentsply DeTrey GmbH, Konstanz, Germany) is the most widely used ERBS and has demonstrated long-term clinical success [18,19]. ERBSs exhibit strong adhesion to dentin and gutta-percha, favorable marginal adaptation, tubular penetration, low solubility, and dimensional stability [20]. However, AH Plus lacks bioactivity and osteogenic potential and has been associated with cytotoxicity, potential genotoxicity, inflammatory responses, hydrophobicity, and material shrinkage [21,22,23,24]. Consequently, it is typically recommended in combination with compaction techniques that minimize sealer volume and compensate for shrinkage [17,25].

Calcium silicate-based cements, such as mineral trioxide aggregate (MTA), were initially introduced as root-end filling materials and are widely used in vital pulp therapy, revitalization, and perforation repair due to their biocompatibility and sealing ability [26,27,28]. CSBSs developed for root canal obturation are bioactive and have antibacterial potential, and provide reliable sealing performance [29]. Available as powder/liquid or premixed formulations, they are hydrophilic materials that set in the presence of moisture, though setting predictability depends on composition and root canal humidity [30,31,32].

Cold hydraulic compaction, a single-cone obturation technique, is the recommended approach for CSBSs [33]. This technique involves placing a matched gutta-percha cone within a sealer-rich environment and is simple and time-efficient, although it may be less suitable for complex canal anatomy [34].

Several studies have compared CSBSs and AH Plus using the single-cone technique [21,35,36]. However, this approach may disadvantage AH Plus due to its physicochemical properties, potentially leading to biased conclusions [37]. Given that the primary objective of RCF is to achieve a void-free seal, technique-dependent differences should be carefully interpreted [38].

The aim of this systematic review and meta-analysis is to compare the RCF quality of CSBSs and AH Plus in permanent mature extracted and artificial teeth, assessing percentage volume and area of voids, and exploring the influence of different obturation techniques (single-cone, warm-vertical compaction, and lateral compaction) on void formation.

To date, no similar meta-analysis has been found in the literature. The present study investigated void formation across different root canal filling techniques and different sealers. Additionally, the investigation focused on different regions of the root canal space and included both extracted and artificial teeth.

## 2. Methods

### 2.1. Protocol and Registration

This systematic review and meta-analysis was conducted in accordance with the Preferred Reporting Items for Systematic Reviews and Network Meta-Analyses (PRISMA) guidelines [39] (Supplementary Table S1). It was registered in the Prospectively Registered Systematic Reviews (PROSPERO) database (CRD42024559126). This investigation was conducted within the framework of the translational cycle model [40].

### 2.2. Eligibility Criteria

The PICOS framework was used. The clinical question was: Is there a difference in root canal filling quality between CSBSs and AH Plus sealer?

The PICOS details were as follows:

- Population (P): Permanent mature extracted teeth and artificial teeth.
- Intervention (I): CSBS.
- Comparator (C): AH Plus (epoxy-amine resin).
- Outcome (O): Percentage volume of voids, percentage area of voids.
- Study (S): In vitro studies.

### 2.3. Inclusion Criteria for Studies

Randomized in vitro studies were included. The review encompassed studies that provided descriptions of the population, clinical procedures, sealer materials used, filling techniques, the number of teeth investigated, and detailed explanations of the outcome measures.

### 2.4. Exclusion Criteria for Studies

Studies excluded from this review included in vivo studies (animal and clinical), such as case reports, two-arm interventional studies, cohort studies, and case–control studies, as well as meta-analyses, reviews, conference abstracts, and papers not published in English. Studies were excluded if the RCF technique was not used.

### 2.5. Information Sources and Search Strategy

An electronic literature search was conducted across the following databases: MEDLINE (PubMed), EMBASE, Cochrane Library, and Web of Science. The search key contains three domains: the first domain pertains to the intervention material (CSBS), the second to the material group, and the third to the comparator material (ERBS). The following search keys were used: (Calcium silicate* OR Bioceramic* OR Hydraulic calcium silicate* OR Hydraulic calcium silicate*) AND (sealer* OR root canal* OR canal filling* OR material*) AND (AH Plus* OR Resin based* OR epoxy resin*). The search keys were adapted to the different databases to reach the highest possible number of results. A citation chase was also conducted, but no new articles were found. The gray literature was not investigated; only peer-reviewed articles were included. The systematic search was completed on 1 July 2025. All articles were imported into EndNote 20 for duplicate removal and were organized alphabetically for manual screening. Initially, automatic duplicate removal was conducted in EndNote 20, followed by a manual review to identify any remaining duplicates.

### 2.6. Selection Process

Two independent reviewers (P.K. and O.V.) screened titles, abstracts, and full texts using Rayyan (https://www.rayyan.ai/ accessed on 12 December 2025). Discrepancies were resolved by discussion or, if needed, consultation with a third author (B.K.). Studies meeting the inclusion criteria underwent full-text screening. Full texts were reviewed for eligibility and inclusion in the meta-analysis, and a citation chase was conducted. Inter-rater reliability was assessed using Cohen’s kappa coefficient.

### 2.7. Data Collection/Extraction Process

Two independent observers extracted data into a predefined Excel spreadsheet (P.K. and O.V.). First, data from some articles meeting the inclusion criteria were extracted into an MS Excel spreadsheet. The statisticians (G.A. and B.L.SZ.) verified the form before the data extraction process. No automated tools were used for data extraction.

### 2.8. Data Items

Collected data included publication details (author/year), study type, prepared teeth, treatment and materials used, root canal preparation technique and final instrument size, irrigation solutions (concentration/time/volume), RCF technique, and storage medium when reported. When numerical data were unavailable, values were extracted using the WebPlotDigitizer tool version 5.2 (https://automeris.io/ accessed on 19 November 2025 [41]. Four studies investigated artificial teeth [36,42,43,44]. Table 1 shows the types of investigated artificial teeth.

### 2.9. Risk of Bias (RoB) Assessment

The risk of bias in in vitro dentistry studies was assessed using the QUIN (Quality Assessment Tool for In Vitro Studies) [45]. The QUIN comprises 12 different criteria: 1. Clearly stated aims/objectives and randomization process, 2. detailed explanation of sample size calculation, 3. detailed explanation of sampling technique, 4. description of comparison group, 5. methodology details, 6. operator information, 7. randomization, 8. outcome measurement method, 9. outcome assessor details, 10. blinding, 11. statistical analysis, and 12. presentation of results. Each criterion was scored by clinicians and researchers as follows: adequately specified = 2 points; inadequately specified = 1 point; and not specified = 0 points. Non-applicable criteria were excluded from the calculation.

Scores were summed to obtain the total score for each in vitro study. These scores were used to categorize in vitro studies as high, medium, or lowrisk of bias. The final score was calculated using the following formula:

Final score = (Total score × 100)/(2 × number of criteria applicable)

A final score exceeding 70% indicates a low risk of bias, a score between 50% and 70% indicates a moderate risk of bias, and a score below 50% indicates a high risk of bias.

### 2.10. Effect Measure and Synthesis Methods

Two outcomes were considered: the percentage volume of voids and the area of voids. Mean values and mean differences (MDs), along with their corresponding 95% confidence intervals (CIs), were used as effect size measures. Study-level means and MDs were calculated using the sample size, mean, and standard deviation (SD) extracted from each study. When only quantiles were reported, means and SDs were estimated using the methods proposed by Luo et al. [46] and Shi et al. [47].

A multilevel random-effects meta-analysis was performed to examine the association between the mean percentage volume of voids and potential predictors. The primary predictor was the combined variable of filling technique and material type, which was analyzed across four analytical settings (Table 2, lines A1–B2). In analysis group A, all CSBSs were compared with the ERBS AH Plus. In analysis group B, the specific CSBS EndoSequence was compared with AH Plus. Root canal regions were incorporated into models A2 and B2 by dividing the volume into apical, middle, and coronal regions, whereas models A1 and B1 did not account for the regional distribution of void volume. Additional predictors included tooth type (extracted vs. artificial) and filling technique (warm vertical compaction vs. matched single cone). For the latter, categories were retained only when enough studies were available, resulting in three analyzable e groups: single-cone technique with CSBSs, and both single-cone and warm vertical compaction techniques with the resin-based AH Plus. Only two studies used AH Plus with the lateral compaction technique; one reported total volume, and the other one reported segmented volume; therefore, this group was not included in the volume analysis.

Mean percentage volume of void values was log-transformed to reduce skewness and stabilize variance, thereby improving model fit and robustness of regression estimates. Several studies reported results for multiple material or design combinations, thereby introducing correlation among the random-effect terms within each study. To account for this, we conducted analyses using the rma.mv() function of the *metafor* R package (version 4.8.0) https://www.metafor-project.org accessed on 10 November 2025, specifying a nested random-effects structure to model within-study dependence. As a sensitivity analysis, a variance–covariance matrix assuming an internal correlation of 0.6 was constructed for models including region (apical, middle, and coronal) as a predictor to account for sampling error correlation. As only marginal differences were observed between model estimates, results from the simpler model are presented.

In analysis C (Table 2, line C), the mean difference in the percentage area of voids was investigated with the lateral compaction technique. The results were investigated at the apical, middle, and coronal levels of the root canal system. Mean differences were calculated with 95% confidence intervals. For these MD calculations, the mean values in the CSBS group were subtracted from those in the resin-based group. Pooled estimates were generated using inverse-variance random-effects models when at least three studies contributed data. Between-study variance (τ^2^) was estimated using the restricted maximum-likelihood method, and statistical heterogeneity was quantified using the I^2^ statistic. Confidence intervals for τ^2^ were obtained using the Q-profile method [48].

All statistical analyses followed the recommendations of Harrer et al. [49] and were performed using R (version 4.4.1). Available online: https://www.R-project.org/ accessed on 10 November 2025). The *meta* package (Version 7.0.0) GitHub Version 7.0.0. https://github.com/guido-s/meta, accessed on 12 December 2025) was used for basic meta-analytic calculations and visualization, while the *dmetar* package (Version 0.1.0) https://dmetar.protectlab.org) was used for additional influence diagnostics and plots. Apart from the meta-regression analyses, all analyses were conducted within the MetaBoostR framework [50].

Publication bias was not formally assessed. Funnel plots and Egger’s regression were considered inappropriate because each synthesis included a limited number of independent studies, and the primary analyses were based on multilevel meta-regression models accounting for correlated effect sizes within studies. Conventional methods for assessing funnel plot asymmetry assume independent effect estimates and have limited validity under these conditions. Furthermore, the Harrer Handbook recommends against routine use of these methods when fewer than 10 studies are available due to their low statistical power and potential for misleading results [49,51].

### 2.11. Certainty Assessment

The certainty of evidence was evaluated by two reviewers (P.K. and O.V.) using the Grading of Recommendations Assessment, Development and Evaluation (GRADE) approach and the GRADE-PRO website (https://www.gradepro.org/ accessed on 9 March 2026). Any disagreements between the reviewers were resolved by a third reviewer (B.K.).

## 3. Results

### 3.1. Study Selection

**Figure 1:**
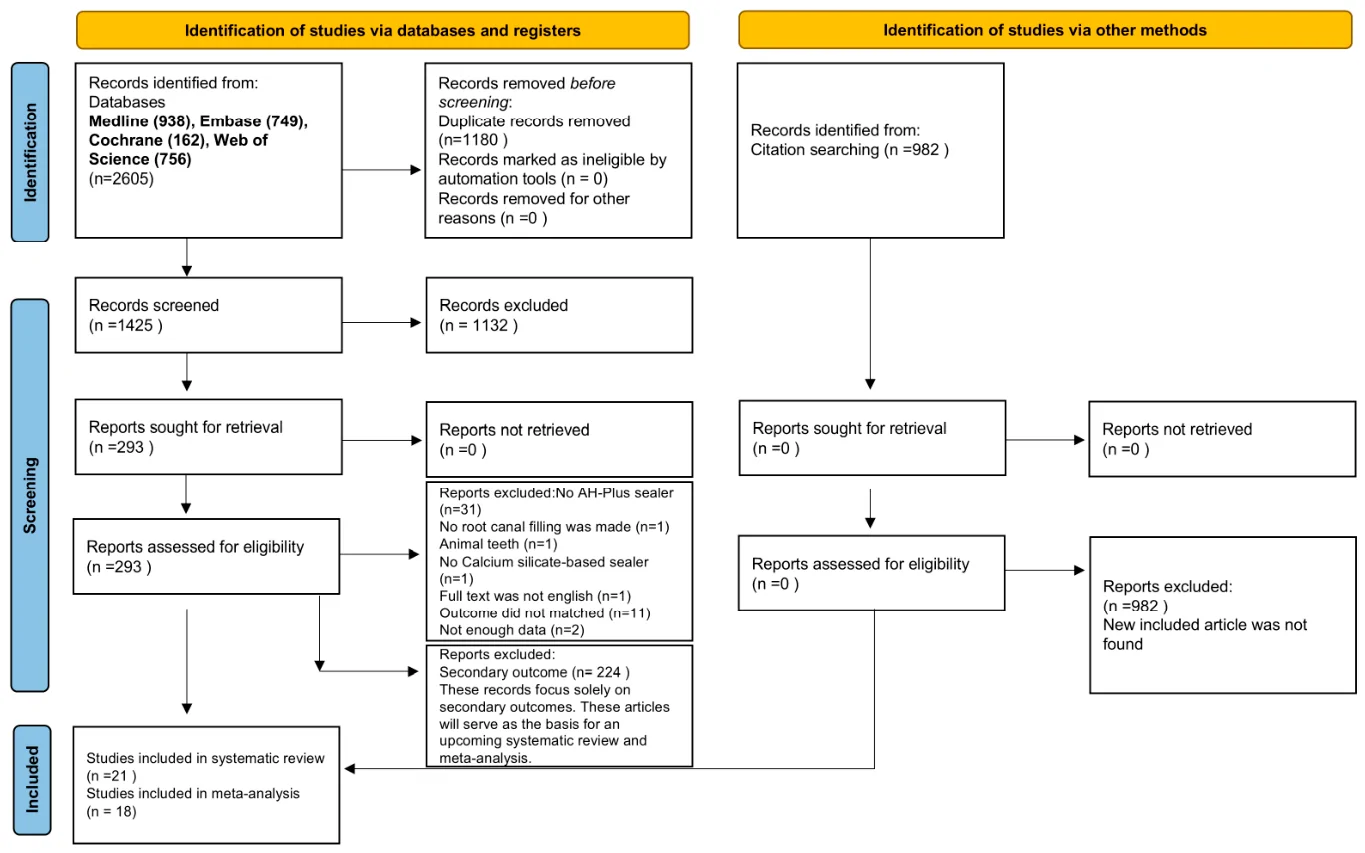
Prisma 2020 flow diagram of the screening and selection process.

The systematic search identified 2605 records in MEDLINE (938), EMBASE (749), CENTRAL (162), and Web of Science (756). After automatic and manual duplicate removal, 1425 articles remained. After title and abstract selection, 293 eligible articles remained. Cohen’s kappa was 0.94 between the two independent observers. After full-text review, 21 articles were selected for inclusion. Cohen’s kappa of the full-text selection was 0.89 (Figure 1). A total of 18 articles were included in the statistical analysis [29,34,35,36,42,43,44,52,53,54,55,56,57,58,59,60,61,62], while three additional articles were used in the systematic review part [14,63,64], yielding a total of 21 included articles.

### 3.2. Study Characteristics

Baseline characteristics of the 21 included studies are presented in Supplementary Table S2.

### 3.3. Results of the Syntheses

Analysis A1. Comparison of the total volume of voids between all CSBSs and AH Plus.

**Figure 2:**
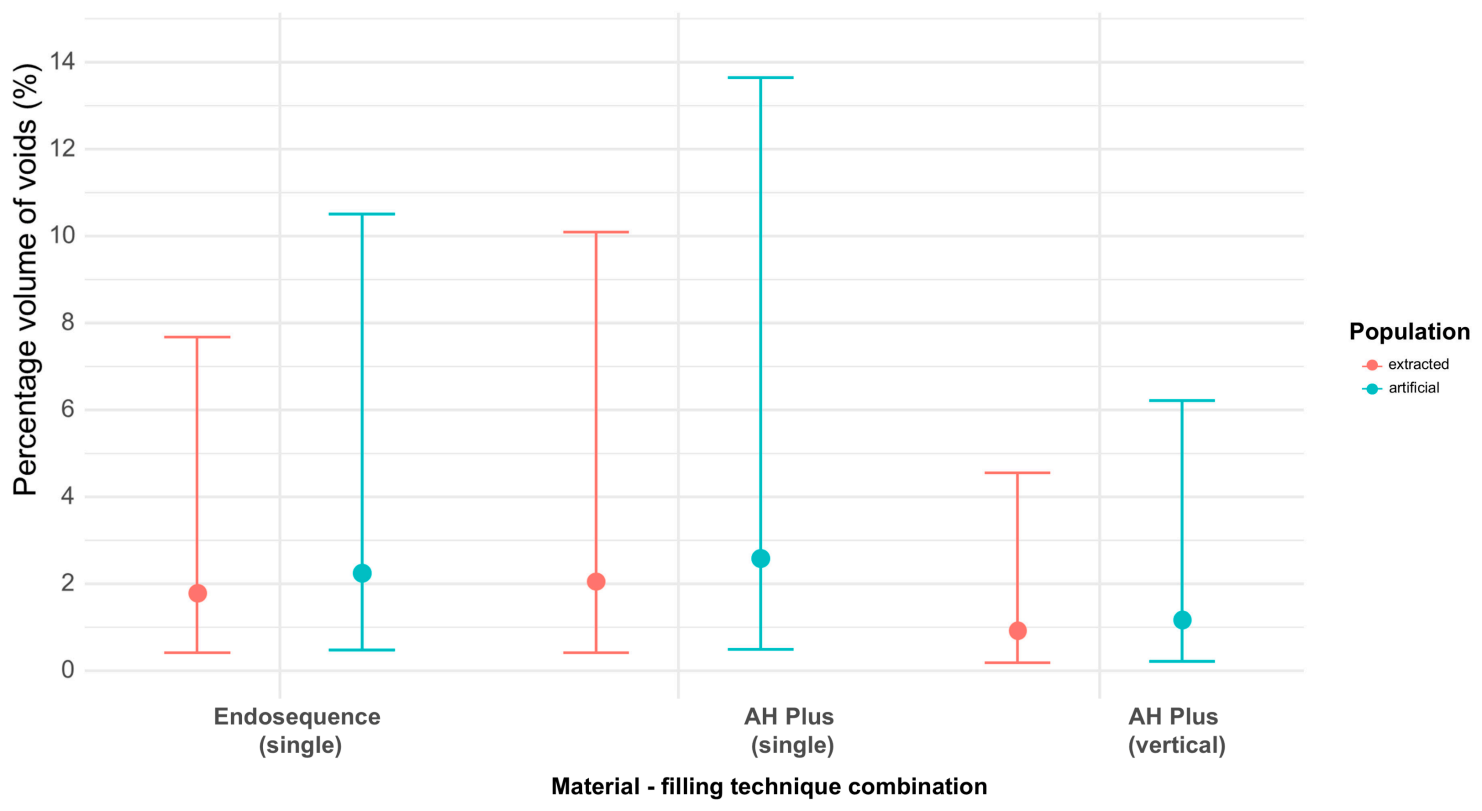
Analysis A1. All CSBSs versus resin-based AH Plus in terms of total volume using single-cone and warm-vertical compaction RCF techniques. The figure presents estimated mean values with 95% confidence intervals for the total volume of the RCF. Red lines represent the extracted teeth population, and blue lines represent the artificial teeth population.

The total number of teeth was 410 across 11 studies [29,36,42,43,44,53,54,55,56,57,58]. The lowest mean value and the narrowest confidence intervals were observed in the AH Plus (vertical) groups (Figure 2, Table 3, and Supplementary Table S3). Artificial teeth yielded results comparable to those of extracted teeth (*p* = 0.5365). No significant differences were found between CSBS (single) and AH Plus (single) (*p* = 0.7237), between CSBS (single) and AH Plus (vertical) (*p* = 0.1204).

Analysis A2. Comparison of the volume of voids in three regions between all CSBSs and AH Plus.

**Figure 3:**
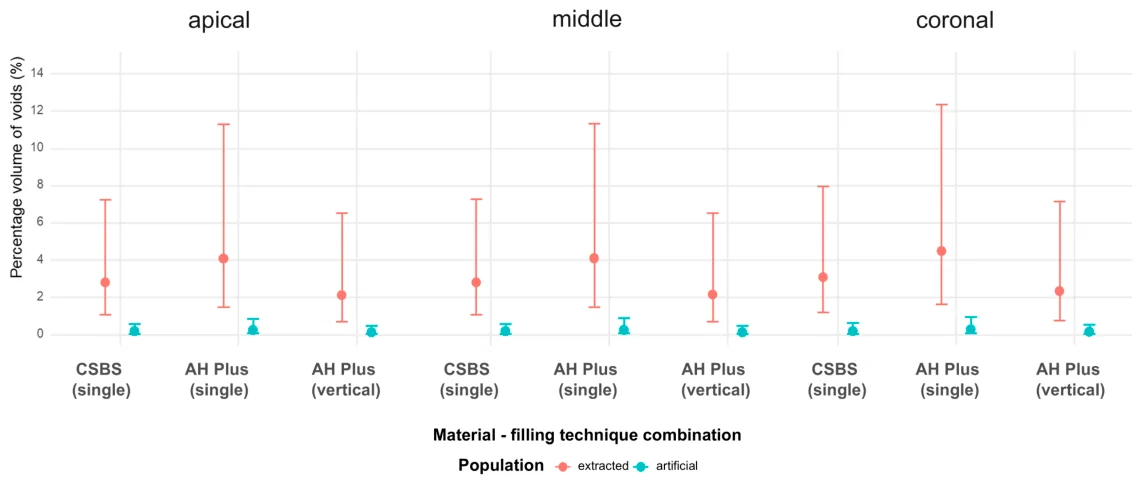
Analysis A2. All CSBSs versus resin-based AH Plus across three regions (apical, middle and coronal regions) using single-cone and warm-vertical compaction RCF techniques. The figure presents estimated mean values with 95% confidence intervals for total RCF volume. Red lines represent the extracted teeth population, and the blue lines represent the artificial teeth population.

The total number of teeth included was 276 across 10 studies [29,34,35,36,42,43,52,56,58,59]. The AH Plus (vertical) compaction showed the lowest mean value in all three regions (apical, middle, and coronal) with extracted teeth (Figure 3). Among the regions, the apical region had the lowest mean value, followed by the middle and coronal regions. There was no significant difference between CSBS (single) and AH Plus (single) (*p* = 0.1268), between CSBS (single) and AH Plus (vertical) (*p* = 0.4376) in extracted teeth in all three regions (Table 4). The artificial teeth group showed significantly lower mean values in all three regions and for all RCF techniques (*p* < 0.001). The void volume in the coronal region did not differ significantly from that in the apical (*p* = 0.6860) and middle regions (*p* = 0.6964) (Supplementary Table S4).

Analysis B1. Comparison of the total volume of voids between EndoSequence CSBS and AH Plus Sealers.

**Figure 4:**
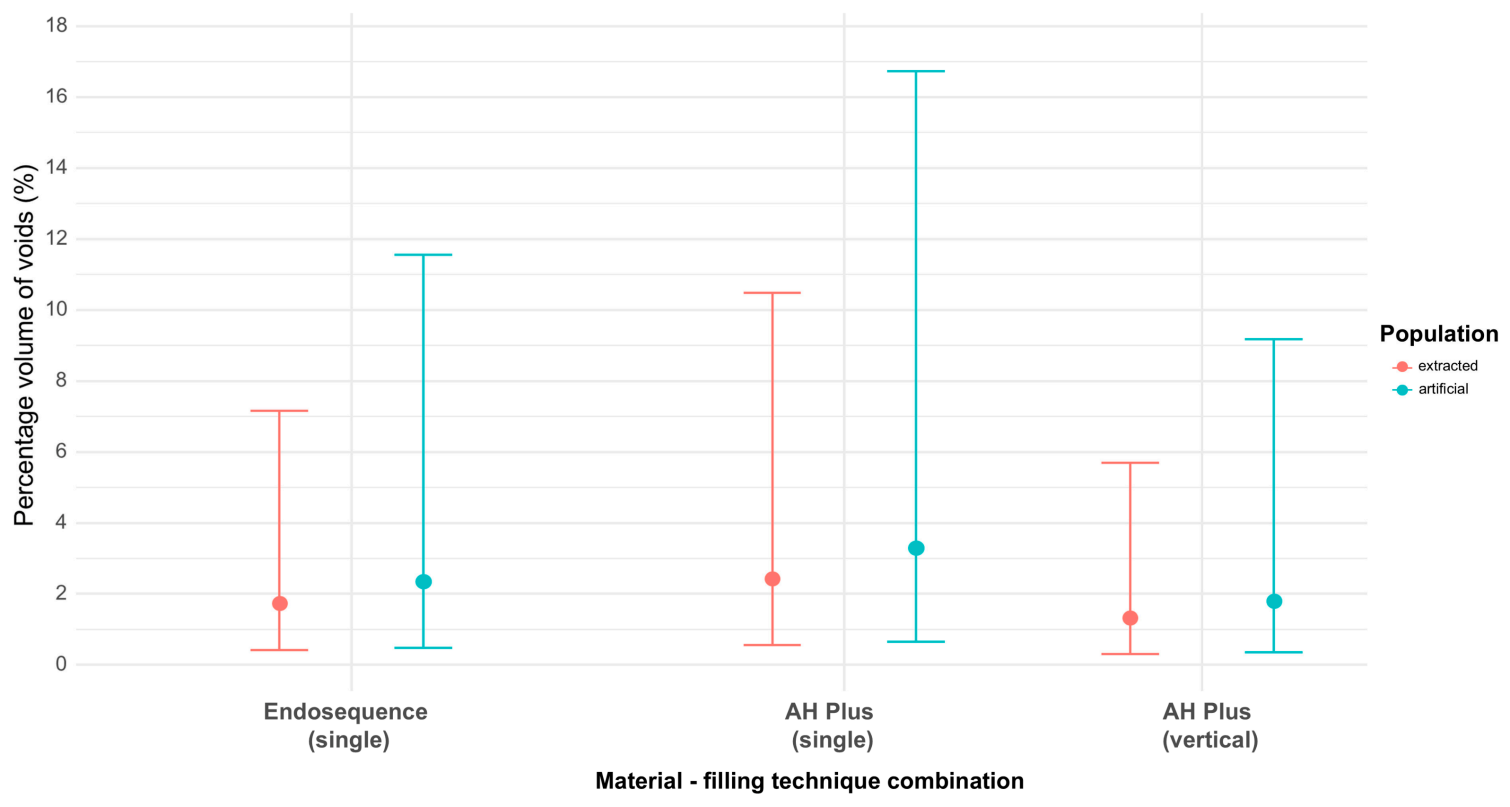
Analysis B1. EndoSequence CSBS versus resin-based AH Plus in total volume with single-cone and warm-vertical compaction RCF techniques. The figure presents estimated mean values with 95% confidence intervals for the total volume of the RCF. Red lines represent the extracted teeth population, and the blue lines represent the artificial teeth population.

A total of 150 teeth were included in six studies [36,44,54,55,56,58]. The lowest mean value and the narrowest confidence intervals were observed for AH Plus (vertical) (Figure 4). Results were comparable between artificial and extracted teeth (*p* = 0.5803) (Table 5 and Supplementary Table S5). There was no significant difference between CSBS (single) and AH Plus (single) (*p* = 0.5584), or between CSBS (single) and AH Plus (vertical) (*p* = 0.6651).

Analysis B2. Comparison of the volume of voids across three regions between EndoSequence CSBS and AH Plus sealers.

**Figure 5:**
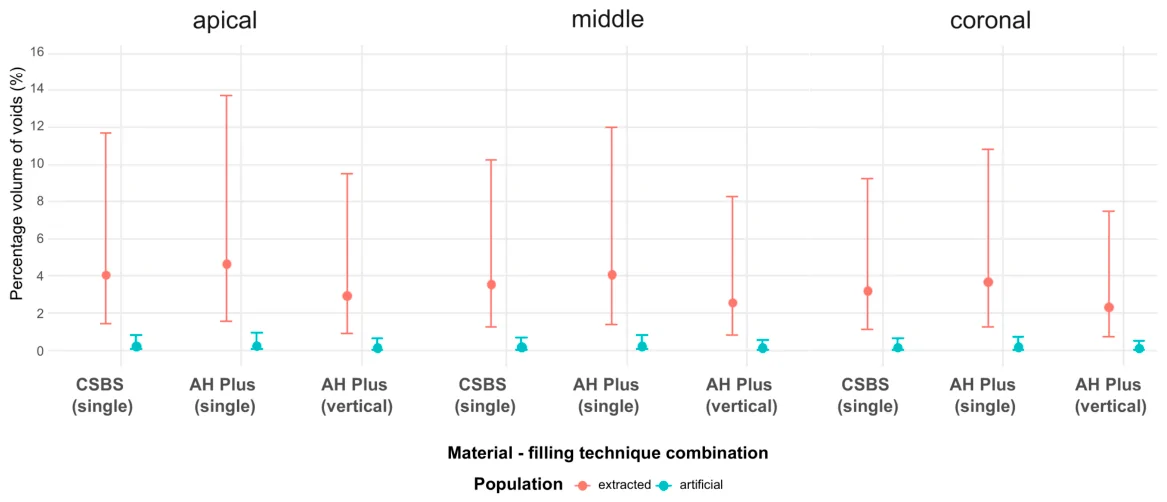
Analysis B2. EndoSequence CSBS versus resin-based AH Plus in three regions (apical, middle, and coronal regions) with single-cone and warm-vertical compaction RCF techniques. The figure presents estimated mean values with 95% confidence intervals in the total volume of the RCF. Red lines represent the extracted teeth population, and the blue lines represent the artificial teeth population.

A total of 94 teeth were included across six studies [34,36,52,56,58,59]. In extracted teeth, AH Plus (vertical) showed the lowest mean value in all three regions (apical, middle, and coronal) (Figure 5). The coronal region showed the lowest mean value across the middle and apical regions. There was no significant difference between CSBS (single) and AH Plus (single) (*p* = 0.6465), or between CSBS (single) and AH Plus (vertical) (*p* = 0.4267), in extracted teeth in all three regions (Table 6, and Supplementary Table S6). All artificial teeth groups showed significantly lower mean values (*p* < 0.001) in all regions than in the extracted teeth groups. The void volume in the coronal region did not differ significantly from that of the apical (*p* = 0.3445) and middle regions (*p* = 0.6717).

Analysis C. Comparison of the area of voids between all CSBSs versus resin-based AH Plus (lateral compaction).

**Figure 6:**
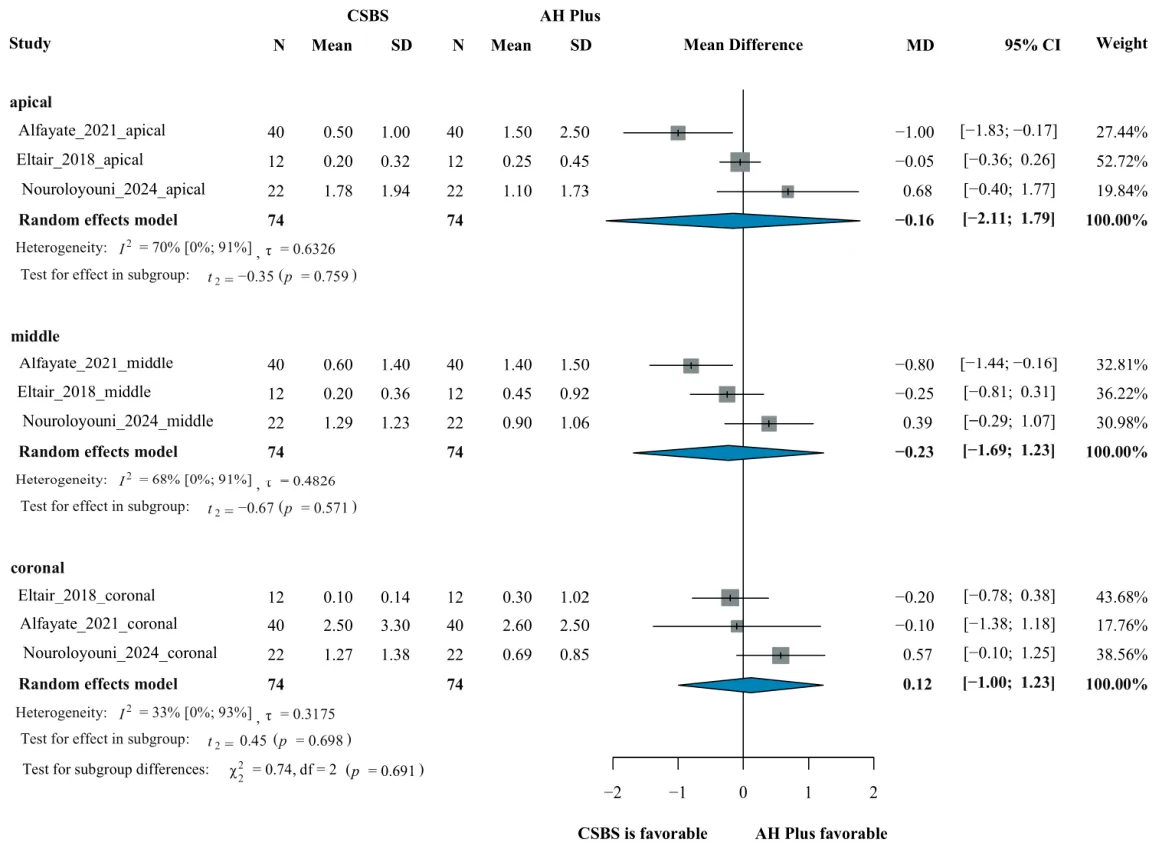
Analysis C. Comparison of the area of voids between all CSBSs versus resin-based AH Plus (lateral compaction). The forest plot illustrates the area of voids across three different sections of the root canal. The plot presents the mean differences between groups, along with their corresponding 95% confidence intervals, allowing comparison of the void distribution across the evaluated sections. The grey squares represent the mean difference (MD), and the length of the black lines indicates the 95% confidence interval (CI). The blue diamonds shows the combined overall result (pooled effect) of all studies. The center point the diamond represents the combined mean difference result (MD) and the width of the diamond indicates the 95% confidence interval (CI) [60,61,62].

The total number of teeth was 148. The number of included studies was 3 [60,61,62]. Mean difference in the apical section was −0.16 [−2.11–1.79]. Mean difference in the middle section was −0.23 [−1.69–1.23]. Mean difference in the coronal section was 0.12 [−1.00–1.23]. The results are not statistically significant in all three levels (Figure 6).

Numerous potential predictors were identified across the included studies. However, the analysis was limited to the most clinically relevant variables: sealer material type, obturation technique, root canal region, and tooth type. Restricting the model to these key predictors enhanced interpretability, although this approach may have left some sources of variability unexplained. The non investigated variables can be categorized into several groups, namely tooth-related characteristics, root canal preparation techniques, irrigation protocols, micro-computed tomography (micro-CT) setting parameters, operator-related factors, storage media, and material properties and these variables contributed to increased heterogeneity.

The results are visualized in ring diagrams to facilitate interpretation for clinicians (Supplementary Figure S1).

### 3.4. Risk of Bias Assessment

One study showed high risk [44], fourteen had some medium risk [14,35,36,42,43,53,54,56,58,59,60,61,63,64], and six indicated low risk [29,34,52,55,57,62] (Table 7).

### 3.5. Certainty of Evidence

The certainty of evidence was moderate across all outcomes (Supplementary Figure S2). The results were consistent across studies. The applicability of the evidence to the review question was examined, and no reason was found to downgrade. Investigating imprecision did not identify a reason for downgrading. The main reason for downgrading was the risk of bias in the included articles. A formal assessment of publication bias was not performed. Therefore, the certainty of evidence was evaluated using a modified GRADE approach. As all included studies were in vitro, the certainty ratings reflect confidence in laboratory findings rather than clinical effectiveness. Consequently, even laboratory evidence rated as high certainty should be interpreted with caution and requires validation through rigorously designed clinical studies.

## 4. Discussion

The present meta-analysis compared CSBSs placed with cold hydraulic compression with the ERBS AH Plus across different RCF techniques (single-cone obturation, warm-vertical compaction, and lateral compaction). Across all pooled analyses, no statistically significant differences were detected between groups in the assessed indicators of filling quality (i.e., void-related outcomes).

Because the included evidence is derived from in vitro studies, the clinical implications of the observed void-related outcomes remain uncertain. Notably, a clinically established threshold for an acceptable void percentage has not yet been defined, which limits the direct translation of small quantitative differences to treatment success. Across the included studies, the estimated mean void percentages in extracted teeth generally ranged from approximately 1% to 5%, depending on the sealer, obturation technique, and root canal region. Although AH Plus, when used with warm vertical compaction, consistently demonstrated the lowest estimated mean void percentages, the pooled analyses did not detect statistically significant differences among the investigated sealer–technique combinations. Therefore, AH Plus was used as a clinically validated reference comparator rather than as a void-free benchmark, reflecting its substantial long-term clinical evidence in endodontic therapy [65].

Warm-vertical compaction requires longer working time, a complex clinical workflow, and greater training than the single-cone technique [66,67], and may increase the risk of overfilling in wide apical foramina [4]. Clinicians should choose the technique based on clinical and anatomical conditions, noting that RCF is only one step of endodontic therapy; consequently, the time saved with the single-cone technique may have only a limited impact on the overall treatment duration.

ERBSs undergo volumetric shrinkage during polymerization. Consequently, obturation techniques that minimize sealer volume—such as lateral compaction or warm vertical compaction—are generally considered more favorable for these materials. In contrast, the tapered single-cone technique relies on a single gutta-percha cone and typically involves a greater volume of sealer, which may theoretically be less advantageous for ERBSs. Despite this expectation, the present meta-analysis did not detect statistically significant differences between warm-vertical compaction and the single-cone techniques, regardless of sealer, in total void volume or in void distribution within any root canal region. The results remained unchanged when the analysis was restricted to studies using EndoSequence as the CSBS in the intervention group. Other CSBS subgroups could not be created due to insufficient number of studies. Similarly, AH Plus used with lateral compaction did not show significantly fewer areas of voids than CSBS-based obturation in any root canal region.

Calcium silicate-based sealers are often grouped together despite notable differences in composition and physicochemical properties. A summary of the composition of the investigated CSBSs is included.

EndoSequence BC Sealer (Brasseler, Savannah, GA, USA) is a premixed, injectable, hydrophilic calcium silicate-based root canal sealer that sets in the presence of moisture within dentinal tubules. It consists primarily of calcium silicates, zirconium oxide, calcium phosphate monobasic, calcium hydroxide, and thickening agents. The material shows high biocompatibility, bioactivity, dimensional stability, and slight expansion during setting, which may enhance the seal of the root canal system. Other commercially available products share the same composition. Safety data sheets indicate that TotalFill (FKG Dentaire, Le Crêt-du-Locle, Switzerland) and iRootSp (Innovative BioCeramix Inc., Burnaby, BC, Canada) possess identical formulations. These materials were therefore analyzed collectively as the same material within the EndoSequence group.

EndoSequence BC HiFlow is a modified version of EndoSequence BC Sealer specifically developed for warm obturation techniques. Unlike the original formulation, HiFlow maintains its physicochemical properties after exposure to elevated temperatures during thermoplastic obturation. It demonstrates excellent flowability, biocompatibility, and bioactivity while maintaining adequate sealing ability under heat. BioRoot RCS (Septodont, Saint-Maur-des-fossés Cedex, France) is a powder-liquid calcium silicate-based sealer. The powder contains tricalcium silicate, zirconium oxide, and povidone, while the liquid mainly consists of calcium chloride and a water-soluble polymer.

BioRoot RCS has demonstrated excellent bioactivity, calcium ion release, alkaline pH, and the ability to stimulate mineralized tissue formation and to achieve a fast setting time. It is one of the most extensively investigated bioactive sealers and has shown favorable biological and sealing properties.

EndoSeal MTA (Maruchi, Vonju, Republic of Korea) is a premixed pozzolan-based calcium silicate sealer containing mineral trioxide aggregate (MTA). The material is characterized by high flowability, short setting time, and excellent penetration into accessory canals. EndoSeal MTA releases calcium ions, promotes biomineralization, and demonstrates favorable sealing ability and biocompatibility. Because of its high flow, careful obturation is recommended to minimize the risk of apical extrusion.

MTA Fillapex (Angelus, Londrina, Brazil) is a root canal sealer containing mineral trioxide aggregate (MTA), salicylate resin, silica nanoparticles, and radiopacifiers. Despite the inclusion of MTA, its properties differ from those of pure calcium silicate sealers because of its resin-based component. Laboratory studies have demonstrated that MTA Fillapex provides satisfactory flow and calcium ion release; however, it also exhibits higher solubility, lower bond strength, and greater cytotoxicity than newer calcium silicate-based sealers (CSBSs).

Bio-C Sealer (Angelus, Londrina, Brazil) is a premixed, ready-to-use calcium silicate-based root canal sealer containing calcium silicates, calcium aluminate, calcium oxide, zirconium oxide, silicon dioxide, and dispersing agents. It is highly bioactive, releases calcium ions, and forms hydroxyapatite upon contact with tissue fluids. Bio-C Sealer demonstrates favorable physicochemical properties, including good flowability, dimensional stability, alkaline pH, and excellent biocompatibility.

Smartpaste Bio (Innovative BioCeramix Inc. Burnaby, BC, Canada) is a premixed CSBS. Its composition includes calcium silicates, calcium phosphate, zirconium oxide, and additional proprietary fillers. The material is engineered to release calcium ions, facilitate hydroxyapatite formation, and establish a biologically favorable seal. Smartpaste Bio demonstrates high biocompatibility and antibacterial activity, which are attributed to its alkaline pH.

iRoot SP (Aibang Technology Company Limited, Shenyang, China) consists primarily of calcium silicates, calcium phosphate, zirconium oxide, and calcium hydroxide. This material is hydrophilic, bioactive, and demonstrates excellent sealing ability due to slight setting expansion and hydroxyapatite formation. Because the exact composition could not be determined, it was analyzed as a separate CSBS in this investigation. Variations in composition, physicochemical properties, and handling characteristics among commercially available CSBSs may affect the formation of voids and clinical performance.

Mechanical preparation alters canal morphology along the apical, middle, and coronal regions [68], potentially affecting RCF quality. The apical portion often has more circular cross sections and, matched to the master gutta-percha cone, may allow better adaptation, whereas the middle and coronal thirds are more irregular, which may compromise adaptation. However, the present meta-analysis did not detect significant differences in void formation between the apical, middle, and coronal portions of the root canal. Celiktan et al. [52] described that these voids are more closely associated with root canal anatomy than with RCF material or technique. Sealer solubility during storage may also contribute to similar void formation.

Three studies were excluded from the quantitative meta-analysis [14,63,64]. Gandolfi et al. [63] investigated the Thermafil obturation technique, which fell outside the predefined technique subgroups of the present analysis. In the studies by Viapiana et al. [14] and Haridas et al. [64], although the intervention groups included CSBSs, the control group used lateral compaction, and the outcome measure was void volume, corresponding to the outcome evaluated in Analysis A. However, because only two studies evaluated this comparison, the available datasets were insufficient for reliable statistical pooling. These studies were therefore excluded from the quantitative synthesis but were considered in the qualitative part of the systematic review.

Gandolfi et al. [63] compared the CSBS MTA-Flow with the ERBS AH Plus using the Thermafil technique, which heats a prefabricated obturator without gutta-percha compaction, potentially limiting adaptation [69]. Micro-CT analysis showed fewer apical voids with MTA-Flow, while the middle and coronal thirds showed no differences. Although excluded from the meta-analysis due to the obturation method, the study provides qualitative evidence on CSBS performance compared with ERBSs.

Viapiana et al. [14] and Haridas et al. [64] investigated CSBSs using the lateral compaction technique. Viapiana et al. [14] found lower void volumes with AH Plus compared to BioRootRCS, whereas Haridas et al. [64] reported lower void volumes with EndoSequence CSBS and BioRootRCS than with AH Plus. Both studies measured voids as a percentage of total filling volume, limiting regional analysis. Despite using the same technique, their results differed, highlighting the need for further standardized studies.

The present study also compared results obtained from extracted teeth and artificial teeth. In the analysis evaluating the total void volume (groups A1 and B1), artificial teeth produced results comparable to those observed in extracted teeth, although the confidence intervals were slightly wider. However, the number of available datasets involving artificial teeth was limited, and these findings should therefore be interpreted with caution. In contrast, in the analyses assessing void volume in three root canal regions, the artificial tooth groups demonstrated significantly lower void percentages than the extracted tooth groups. The amount of available data in this subgroup was even more limited; these results should be interpreted with additional caution.

Four articles with artificial teeth were included in the investigation [36,42,43,44]. For the total volume analyses (groups A1 and B1), only one study used artificial teeth [44]. In contrast, the A2 analysis included three studies [36,42,43] with a larger overall sample size. Similarly, only one study [36] was included in the B2 analysis. This study also had a relatively small sample size, and the mean volume percentage was low, with narrow confidence intervals. Another possible explanation for these findings may be the type of artificial teeth used. In the A1 and B1 analyses, extracted teeth were scanned and reproduced using three-dimensional printing. In the remaining studies, commercially manufactured artificial teeth or plastic blocks were used. Structural differences between artificial teeth and extracted human teeth may influence sealer behavior during obturation, potentially affecting void formation and the overall quality of the root canal filling.

### 4.1. Limitations

Several limitations of the present study should be acknowledged. First, all included studies were conducted in vitro, which limits the direct clinical applicability of the findings. In addition, methodological details such as operator characteristics, outcome assessor information, and sample blinding were not reported in most studies, potentially introducing bias and heterogeneity.

Second, the included studies contain data about tooth anatomy, tooth group of extracted teeth (anterior, premolar or molar), and canal morphology. Root canal preparation protocols were generally reported in considerable detail. Most studies described the shaping technique, the instrumentation system used, and the size of the master apical file. In addition, irrigation protocols, including the irrigants and their application procedures, were documented. Micro-CT image set-up parameters and analysis, such as scanning resolution, reconstruction parameters, and image processing protocols, may also have introduced variability between studies. Because of the high number of predictors, the study focuses on the most important predictors: the sealer material type, obturation technique, root canal region, and tooth type (extracted or artificial). The not-evaluated predictor may introduce heterogeneity.

Third, the formation of voids in RCFs can be affected by operator experience, sealer properties, and obturation technique. Studies used various storage media (thymol, distilled water, phosphate-buffered saline), whose effects on materials or tooth structure were not systematically evaluated. These uncontrolled factors may have contributed to variability and wide confidence intervals in the meta-analysis.

Fourth, in one study [56], numerical data were not provided in tabulated form and therefore had to be extracted from figures using the WebPlotDigitizer tool (Ankit Rohatgi, https://automeris.io, version 5.2 accessed on 19 November 2025). In addition, some studies reported median values rather than means and standard deviations. For these studies, mean values and standard deviations were estimated using the methods proposed by Luo et al. [46] and Shi et al. [47]. This approach was applied to the studies by Milanovic et al. [56] and Wisawawatin et al. [34], which may introduce additional uncertainty into the analysis.

Fifth, while the presence of voids is considered an important indicator of obturation quality, it constitutes only one surrogate outcome. Other clinical factors may also influence the long-term clinical success of endodontically treated teeth.

### 4.2. Strengths

The meta-analysis included a relatively large sample size of extracted and artificial teeth (*n* = 656) and a substantial number of studies (*n* = 18) for statistical analysis. The sample size is the total number of extracted and artificial teeth included. The sample sizes of individual analyses may vary depending on the included studies.

In addition, the study provides a comprehensive comparison of RCF quality between AH Plus and CSBSs across different obturation techniques. Both extracted and artificial teeth were included, allowing a broader evaluation of experimental models used in endodontic research. Furthermore, most of the included studies (*n* = 15) assessed void formation using micro-computed tomography (micro-CT), which provides high-resolution three-dimensional data and is considered a reliable method for evaluating the quality of RCFs. Finally, the study follows the principles of the translational cycle model, which aims to facilitate the clinical application of research findings and supports the integration of scientific evidence into clinical dentistry [40].

### 4.3. Implication for Research/Clinical

#### 4.3.1. Research Implications

Further well-designed in vitro studies are needed to assess RCF quality across different sealers and obturation techniques. Micro-CT provides high-resolution three-dimensional data, allowing precise localization of voids.

Future studies should aim to improve methodological transparency by providing detailed descriptions of operator characteristics, outcome assessor information, and sample blinding procedures. In addition, standardized experimental protocols should be considered, including the selection of storage media that do not interfere with the physical properties of dental hard tissues or filling materials.

Further investigations of the lateral compaction technique are also warranted. Despite its long-standing clinical use, ease of application, and cost-effectiveness, only a limited number of micro-CT studies have evaluated its performance compared with other obturation techniques.

Finally, the use of artificial teeth in in vitro studies warrants further investigation. Based on the limited data available from this analysis, the results from artificial teeth should be interpreted with caution until more evidence becomes available.

#### 4.3.2. Clinical Implications

Cold hydraulic compaction (single-cone technique) is currently the most widely recommended obturation method for CSBSs. However, recently introduced CSBS materials may also be used with other RCF techniques. Based on the findings of the present meta-analysis, no significant differences in void formation were observed between the investigated techniques, suggesting that CSBSs may provide comparable filling quality across different obturation approaches.

CSBSs may provide comparable filling quality regardless of the obturation technique used, suggesting that they can achieve an adequate seal across different clinical approaches. This versatility may allow clinicians greater flexibility when selecting an obturation method without compromising the overall quality of the root canal filling.

One of the major advantages of CSBSs is their excellent biocompatibility. This property is particularly valuable in clinical situations where the sealer is likely to come into direct contact with surrounding vital tissues, such as apical extrusion, root perforations, or resorptive defects. Their favorable biological response may reduce tissue irritation and promote healing, making them an attractive option in challenging endodontic cases. Consequently, these clinical considerations may play an important role in the clinician’s choice of sealer.

Clinical situations with an increased risk of overfilling may also represent suitable indications for the use of CSBSs. Unlike some conventional sealers, calcium silicate-based materials generally exhibit favorable tissue compatibility, which may lessen the biological consequences of unintended extrusion beyond the apical foramen.

Similarly, CSBSs may be particularly advantageous for managing strip perforations due to their bioactive properties, sealing ability, and potential to support hard tissue formation. These characteristics can contribute to improved healing and a more favorable long-term prognosis.

Despite these advantages, CSBSs may pose challenges during nonsurgical endodontic retreatment. Their strong adhesion to dentinal walls and hard structure can make complete removal from the root canal system difficult. As a result, clinicians should carefully consider the potential need for future retreatment when selecting these materials.

Nevertheless, further well-designed in vitro studies and clinical investigations are required to confirm these findings under clinical conditions.

The synthesis of individual study results through meta-analysis can contribute to the development of high-level evidence and support evidence-based clinical decision-making [70]. In this context, translational frameworks such as the ring diagram may facilitate the implementation of scientific findings into everyday clinical practice [71].

## 5. Conclusions

The present meta-analysis found no statistically significant differences in void formation between calcium silicate-based sealers and AH Plus when used with the investigated root canal filling techniques. Voids were observed across all sealer–technique combinations, indicating that some degree of void formation is inherent to root canal obturation under in vitro conditions. Because no clinically acceptable threshold for void formation has been established, the clinical relevance of the observed differences remains uncertain. The available evidence is limited by methodological heterogeneity and is derived primarily from in vitro studies. Further standardized laboratory investigations and well-designed clinical studies are needed to establish the clinical significance of void formation and to determine whether specific sealer–technique combinations provide superior long-term outcomes.

## Figures and Tables

**Table 1:** Investigated artificial teeth in the included studies.

Study with Artificial Teeth	Type of Artificial Teeth
Kim et al. 2020 [42]	Simulated root canals in the resin blocks of SCS-SP181 (SCS, Foshan, China) with a J-shaped curve and a mean canal length of 18.0 mm.
Lee et al. 2022 [43]	Forty-five artificial mandibular molar teeth (TRUETOOTH #19, DELABS, Santa Barbara, CA, USA) with an isthmus between the mesio-lingual and mesio-buccal canals.
Kooanantkul et al. 2023 [44]	Incisors and mesial roots of lower molars with standardized root length were scanned using μCT, and one tooth of each type was 3D printed in acrylic.
Lin et al. 2023 [36]	Twenty 3D-printed upper first premolars.

**Table 2:** Analysis design.

Analysis	Outcome	Intervention		Comparator			Additional Predictor	
		Sealer	Obturation Mode	Sealer	Obturation Mode			
A1	volume of voids	all CSBSs	single cone	AH Plus	single cone	warm vertical compaction	tooth type	
A2		all CSBSs	single cone	AH Plus	single cone	warm vertical compaction	tooth type	region location
B1		EndoSequence	single cone	AH Plus	single cone	warm vertical compaction	tooth type	
B2		EndoSequence	single cone	AH Plus	single cone	warm vertical compaction	tooth type	region location
C	area of voids	all CSBSs	single cone	AH Plus	lateral compaction			

(A1) Total volume of obturation material comparing all calcium silicate-based sealers and AH Plus using the single-cone technique, with additional comparison to warm-vertical compaction. Tooth type was included as an additional predictor. (A2) Coronal volume of obturation material under the same conditions as (A1), with additional adjustment for region location and tooth type. (B1) Total volume comparison between EndoSequence sealer and AH Plus using the single-cone technique, with warm-vertical compaction as a comparator. Tooth type was included as an additional predictor. (B2) Coronal volume comparison between EndoSequence and AH Plus under the same obturation conditions as (B1), with adjustment for both tooth type and segment location. (C) Area of voids comparing all CSBSs applied with the single-cone technique versus AH Plus used with lateral compaction.

**Table 3:** Analysis A1 This table presents the estimated mean values and corresponding confidence intervals of percentage volume of voids for different obturation materials and techniques across two tooth populations (extracted and artificial). The groups include calcium silicate-based and resin-based (AH Plus) sealers applied using single-cone and warm-vertical root canal filling techniques. Mean estimates are reported alongside corresponding lower and upper confidence interval bounds.

Predictor	Population	Estimated Mean	Confidence Interval Lower Bound	Confidence Interval Upper Bound
CSBS (single)	extracted	1.794	0.419	7.683
AH-Plus (single)	extracted	2.063	0.422	10.098
AH-Plus (vertical)	extracted	0.938	0.193	4.565
CSBS (single)	artificial	2.261	0.486	10.514
AH-Plus (single)	artificial	2.600	0.495	13.646
AH-Plus (vertical)	artificial	1.181	0.225	6.217

**Table 4:** Analysis A2. This table presents estimated mean values and corresponding confidence intervals (lower and upper bounds) of the percentage volume of voids for each predictor group. Predictors include all CSBSs (single-cone obturation technique) and AH Plus (single and warm-vertical compaction obturation techniques). Data are stratified by tooth population (extracted vs. artificial) and root region (coronal, middle, and apical). Mean estimates are reported alongside corresponding lower and upper confidence interval bounds.

Predictor	Population	Region	Estimated Mean	Confidence Interval Lower Bound	Confidence Interval Upper Bound
CSBS (single)	extracted	coronal	3.083	1.193	7.968
AH-Plus (single)	extracted	coronal	4.488	1.629	12.367
AH-Plus (vertical)	extracted	coronal	2.343	0.768	7.149
CSBS (single)	artificial	coronal	0.195	0.061	0.627
AH-Plus (single)	artificial	coronal	0.284	0.084	0.959
AH-Plus (vertical)	artificial	coronal	0.149	0.041	0.544
CSBS (single)	extracted	apical	2.807	1.086	7.254
AH-Plus (single)	extracted	apical	4.086	1.476	11.308
AH-Plus (vertical)	extracted	apical	2.133	0.698	6.517
CSBS (single)	artificial	apical	0.178	0.056	0.567
AH-Plus (single)	artificial	apical	0.259	0.077	0.871
AH-Plus (vertical)	artificial	apical	0.135	0.037	0.493
CSBS (single)	extracted	middle	2.816	1.089	7.280
AH-Plus (single)	extracted	middle	4.099	1.483	11.327
AH-Plus (vertical)	extracted	middle	2.140	0.701	6.532
CSBS (single)	artificial	middle	0.178	0.056	0.570
AH-Plus (single)	artificial	middle	0.260	0.077	0.874
AH-Plus (vertical)	artificial	middle	0.136	0.037	0.495

**Table 5:** Analysis B1 This table presents the estimated mean values and corresponding confidence intervals of the percentage volume of voids for different obturation materials and techniques across two tooth populations (extracted and artificial). The groups include calcium silicate-based and resin-based (AH Plus) sealers applied using single-cone and warm-vertical root canal filling techniques. Mean estimates are reported alongside corresponding lower and upper confidence interval bounds.

Predictor	Population	Estimated Mean	Confidence Interval Lower Bound	Confidence Interval Upper Bound
Endosequence (single)	extracted	1.734	0.420	7.166
AH Plus (single)	extracted	2.433	0.565	10.482
AH Plus (vertical)	extracted	1.335	0.313	5.688
Endosequence (single)	artificial	2.351	0.478	11.557
AH Plus (single)	artificial	3.298	0.650	16.730
AH Plus (vertical)	artificial	1.809	0.357	9.177

**Table 6:** Analysis B2. This table presents estimated mean values and corresponding confidence intervals (lower and upper bounds) of percentage volume of voids for each predictor group. Predictors include all CSBSs (single-cone obturation technique) and AH Plus (single and warm-vertical compaction obturation techniques). Data are stratified by tooth population (extracted vs. artificial) and root region (coronal, middle, and apical). Mean estimates are reported alongside corresponding lower and upper confidence interval bounds.

Predictor	Population	Region	Estimated Mean	Confidence Interval Lower Bound	Confidence Interval Upper Bound
Endosequence (single)	extracted	coronal	3.206	1.113	9.237
AH Plus (single)	extracted	coronal	3.677	1.250	10.812
AH Plus (vertical)	extracted	coronal	2.320	0.721	7.465
Endosequence (single)	artificial	coronal	0.169	0.045	0.635
AH Plus (single)	artificial	coronal	0.194	0.051	0.740
AH Plus (vertical)	artificial	coronal	0.123	0.030	0.494
Endosequence (single)	extracted	apical	4.066	1.414	11.690
AH Plus (single)	extracted	apical	4.662	1.585	13.718
AH Plus (vertical)	extracted	apical	2.942	0.912	9.490
Endosequence (single)	artificial	apical	0.215	0.057	0.804
AH Plus (single)	artificial	apical	0.246	0.065	0.940
AH Plus (vertical)	artificial	apical	0.155	0.039	0.628
Endosequence (single)	extracted	middle	3.563	1.242	10.227
AH Plus (single)	extracted	middle	4.086	1.389	12.016
AH Plus (vertical)	extracted	middle	2.579	0.803	8.281
Endosequence (single)	artificial	middle	0.188	0.050	0.706
AH Plus (single)	artificial	middle	0.216	0.056	0.826
AH Plus (vertical)	artificial	middle	0.136	0.034	0.550

**Table 7:** Risk of bias assessment result according to the QUIN (Quality Assessment Tool for In Vitro Studies).

	Criteria	1. Clearly Stated Aims/Objectives	2. Detailed Explanation of Sample Size Calculation	3. Detailed Explanation of Sampling Technique	4. Details of Comparison Group	5. Detailed Explanation of Methodology	6. Operator Details	7. Randomization	8. Method of Measurement of Outcome	9. Outcome Assessor Details	10. Blinding	11. Statistical Analysis	12. Presentation of Results	Total Score	Final Score	Risk
Study																
**Celikten et al. 2016 [52]**		2	2	2	2	2	1	1	2	1	1	2	2	20	**83** **.** **33**	**low**
**Kim et al. 2018 [53]**		2	0	2	2	2	0	1	2	1	0	1	2	15	**62** **.** **5**	**medium**
**Yanpiset et al. 2018 [54]**		2	0	2	2	2	0	1	2	0	0	1	2	14	**58** **.** **33**	**medium**
**Roizenblit et al. 2020 [55]**		2	2	2	2	2	1	1	2	1	0	2	2	19	**79** **.** **17**	**low**
**Milanovic et al. 2020 [56]**		2	1	2	1	2	0	1	2	0	0	2	1	14	**58** **.** **33**	**medium**
**Tavares et al. 2021 [35]**		2	2	1	2	2	0	2	2	0	0	2	1	16	**66** **.** **67**	**medium**
**Kim et al. 2020 [42]**		2	0	1	2	2	0	1	2	0	0	2	2	14	**58** **.** **33**	**medium**
**Pinto et al. 2021 [57]**		2	2	2	2	2	0	2	2	1	0	2	2	19	**79** **.** **17**	**low**
**Kim et al. 2021 [58]**		2	0	2	2	2	0	1	2	0	0	2	2	15	**62** **.** **5**	**medium**
**Jin et al. 2021 [29]**		2	0	2	2	2	0	1	2	1	1	2	2	17	**70** **.** **83**	**low**
**Lee et al. 2022 [43]**		1	0	2	2	2	1	1	2	0	0	2	2	15	**62** **.** **5**	**medium**
**Kooanantkul et al. 2023 [44]**		2	0	1	1	1	0	0	2	0	0	2	1	10	**41** **.** **67**	**high**
**Lin et al. 2023 [36]**		2	0	1	1	2	0	0	2	0	0	2	2	12	**50**	**medium**
**Wisawawatin et al. 2023 [34]**		2	2	2	2	2	0	2	2	0	0	2	2	18	**75**	**low**
**Li et al. 2025 [59]**		2	0	2	1	2	0	0	2	0	0	2	2	13	**54** **.** **17**	**medium**
Eltair et al. 2018 * [60]		2	0	2	2	2	0	1	2	0	0	2	1	14	**58** **.** **33**	**medium**
Alfayate et al. 2021 * [61]		2	0	2	2	2	0	1	2	0	0	2	1	14	**58** **.** **33**	**medium**
Nouroloyouni et al. 2023 * [62]		2	0	2	2	2	2	1	2	2	1	2	2	20	**83** **.** **33**	**low**
**Gandolfi et al. 2013 # [63]**		2	0	2	2	2	0	1	2	0	0	1	2	14	**58** **.** **33**	**medium**
**Viapiana et al. 2016 # [14]**		2	0	2	1	2	0	0	2	0	0	2	2	13	**54** **.** **17**	**medium**
**Haridas et al. 2024 # [64]**		1	0	2	2	2	0	1	2	0	0	1	2	13	**54** **.** **17**	**medium**

* In the designated articles the published data was the percentage area of voids. # The designated articles were included in to the systematic review only. Final score = (Total score × 100)/(2 × number of criteria applicable (12)). Final score > 70% low risk of bias, 70–50% medium risk of bias, <50% high risk of bias. Criteria as adequately specified = 2 points, inadequately specified = 1 point, not specified = 0 point.

## Data Availability

Data supporting the findings of this study are available from the corresponding author upon reasonable request.

## Supplementary Materials

The following supporting information can be downloaded at: https://www.mdpi.com/article/10.3390/dj14080496/s1, Supplementary Figure S1: Ring Diagram; Supplementary Figure S2: Certanity of evidence; Supplementary Table S1: Prisma 2020 checklist [39]; Supplementary Table S2: Baseline characteristics of included articles; Supplementary Table S3: Estimated coefficients of percentage volume of voids from Analysis A1; Supplementary Table S4: Estimated coefficients of percentage volume of voids from Analysis A2; Supplementary Table S5: Estimated coefficients of percentage volume of voids from Analysis B1; Supplementary Table S6: Estimated coefficients of percentage volume of voids from Analysis B2.

## Abbreviations

The following abbreviations are used in this manuscript:

CI	confidence interval
CSBS	calcium silicate-based sealer
ERBS	epoxy resin-based sealer
MD	mean difference
MTA	mineral trioxide aggregate
PRISMA	Preferred Reporting Items for Systematic Reviews and Network Meta-Analyses
PROSPERO	Prospectively Registered Systematic Reviews
QUIN	Quality Assessment Tool for In Vitro Studies
RCF	root canal filling
