# Supplementary material for: In Vitro Comparison of Void Formation Between Calcium Silicate-Based Sealers and AH Plus: A Systematic Review and Meta-Analysis
Source: Dent J (Basel). 2026 Aug 7;14(8):496. doi: 10.3390/dj14080496 (PMC13511041; doi:10.3390/dj14080496)
Supplement: Supplementary file 1 [file dentistry-14-00496-s001.zip › Supplementary_Table_S3_AnalysisA1_p_value.pdf]

Supplementary Table S3. Estimated coefficients of percentage volume of voids from Analysis A1, including standard errors, p-values, and 95% confidence intervals (CI; lower bound and upper bound). The intercept was the single-cone obturation technique with calcium silicate-based sealer. The p-values belong to the comparison between the intercept group and the resin single-cone, warm-vertical and artificial teeth group.

	**Estimate**	**Standard error**	**p-value**	**Confidence interval lower bound**	**Confidence interval upper bound**
intercept	-4.0207	0.7226	0.0000	-5.475	-2.566
AH-Plus (single)	0.1398	0.3932	0.7237	-0.652	0.931
AH-Plus (vertical)	-0.6489	0.4101	0.1204	-1.474	0.177
Artificial	0.2311	0.3711	0.5365	-0.516	0.978
