# Supplementary material for: In Vitro Comparison of Void Formation Between Calcium Silicate-Based Sealers and AH Plus: A Systematic Review and Meta-Analysis
Source: Dent J (Basel). 2026 Aug 7;14(8):496. doi: 10.3390/dj14080496 (PMC13511041; doi:10.3390/dj14080496)
Supplement: Supplementary file 1 [file dentistry-14-00496-s001.zip › Supplementary_Table_S4_AnalysisA2_p_value.pdf]

Supplementary table S4. Estimated coefficients of percentage volume of voids from Analysis A2, including standard errors, p-values, and 95% confidence intervals (CI; lower bound and upper bound). The intercept represents the baseline condition calcium silicate-based sealer with the single-cone obturation technique and the coronal region.

	**Estimate**	**Standard error**	**p-value**	**Confidence interval lower bound**	**Confidence interval upper bound**
intercept	-3.479	0.477	0.000	-4.429	-2.530
AH-Plus (single)	0.375	0.243	0.127	-0.109	0.860
AH-Plus (vertical)	-0.274	0.352	0.438	-0.974	0.426
Artificial	-2.759	0.476	p<0.001	-3.705	-1.812
Apical	-0.094	0.231	0.686	-0.554	0.366
Middle	-0.091	0.231	0.696	-0.551	0.370
