# Supplementary material for: In Vitro Comparison of Void Formation Between Calcium Silicate-Based Sealers and AH Plus: A Systematic Review and Meta-Analysis
Source: Dent J (Basel). 2026 Aug 7;14(8):496. doi: 10.3390/dj14080496 (PMC13511041; doi:10.3390/dj14080496)
Supplement: Supplementary file 1 [file dentistry-14-00496-s001.zip › Supplementary_Table_S5_AnalysisB1_p_value.pdf]

Supplementary table S5. Estimated coefficients of percentage volume of voids from Analysis B1, including standard errors, p-values, and 95% confidence intervals (CI; lower bound and upper bound). The intercept was the single-cone obturation technique with EndoSequence calcium silicate-based sealer.

	**Estimate**	**Standard error**	**p-value**	**Confidence interval lower bound**	**Confidence interval upper bound**
intercept	-4.055	0.694	0.0000	-5.474	-2.636
AH Plus (single)	0.339	0.572	0.5584	-0.832	1.509
AH Plus (vertical)	-0.262	0.598	0.6651	-1.485	0.962
artificial	0.304	0.544	0.5803	-0.808	1.417
