# Supplementary material for: In Vitro Comparison of Void Formation Between Calcium Silicate-Based Sealers and AH Plus: A Systematic Review and Meta-Analysis
Source: Dent J (Basel). 2026 Aug 7;14(8):496. doi: 10.3390/dj14080496 (PMC13511041; doi:10.3390/dj14080496)
Supplement: Supplementary file 1 [file dentistry-14-00496-s001.zip › Supplementary_Table_S6_AnalysisB2_p_value.pdf]

Supplementary table S6. Estimated coefficients of percentage volume of voids from Analysis B2, including standard errors, p-values, and 95% confidence intervals (CI; lower bound and upper bound). The intercept represents the baseline condition EndoSequence calcium silicate-based sealer with single-cone obturation technique and coronal region

	**Estimate**	**Standard error**	**p-value**	**Confidence interval lower bound**	**Confidence interval upper bound**
intercept	-3.440	0.526	0.0000	-4.498	-2.382
AH Plus (single)	0.137	0.296	0.6465	-0.459	0.733
AH Plus (vertical)	-0.324	0.404	0.4267	-1.135	0.488
Artificial	-2.940	0.550	p<0.001	-4.046	-1.834
Apical	0.237	0.249	0.3445	-0.263	0.738
Middle	0.106	0.248	0.6717	-0.392	0.603
